# Deep learning-based super-resolution US radiomics to differentiate testicular seminoma and non-seminoma: an international multicenter study

**DOI:** 10.1186/s13244-025-02045-y

**Published:** 2025-08-01

**Authors:** Yafang Zhang, Shilin Lu, Chuan Peng, Shichong Zhou, Irene Campo, Michele Bertolotto, Qian Li, Zhiyuan Wang, Dong Xu, Yun Wang, Jinshun Xu, Qinfu Wu, Xiaoying Hu, Wei Zheng, Jianhua Zhou

**Affiliations:** 1 Department of Ultrasound, Sun Yat-sen University Cancer Center, State Key Laboratory of Oncology in South China, Guangdong Provincial Clinical Research Center for Cancer, Guangzhou, China; 2 Department of Ultrasonography, Fudan University Shanghai Cancer Center, Shanghai, China; 3 Department of Radiology, University of Trieste, Gorizia, Italy; 4 Department of Radiology, University Hospital Trieste, Trieste, Italy; 5 Department of Ultrasound, The Affiliated Cancer Hospital of Zhengzhou University and Henan Cancer Hospital, Zhengzhou, China; 6 Department of Medical Ultrasound, Hunan Cancer Hospital/The Affiliated Cancer Hospital of Xiangya School of Medicine, Central South University, Changsha, China; 7 Department of Ultrasound, Zhejiang Cancer Hospital, Hangzhou, China; 8 Department of Medical Ultrasound, Yunnan Cancer Hospital, Kunming, China; 9 Department of Ultrasound, Sichuan Clinical Research Center for Cancer, Sichuan Cancer Hospital & Institute, Sichuan Cancer Center, Affiliated Cancer Hospital of University of Electronic Science and Technology of China, Chengdu, China; 10 Department of Ultrasound, Zhuhai Hospital, Guangdong Hospital of Traditional Chinese Medicine, Zhuhai, China; 11 Department of Ultrasound, Linyi Cancer Hospital, Linyi, China

**Keywords:** Seminoma, Non-seminoma, Radiomics, Super-resolution, Ultrasound

## Abstract

**Objectives:**

Subvariants of testicular germ cell tumor (TGCT) significantly affect therapeutic strategies and patient prognosis. However, preoperatively distinguishing seminoma (SE) from non-seminoma (n-SE) remains a challenge. This study aimed to evaluate the performance of a deep learning-based super-resolution (SR) US radiomics model for SE/n-SE differentiation.

**Materials and methods:**

This international multicenter retrospective study recruited patients with confirmed TGCT between 2015 and 2023. A pre-trained SR reconstruction algorithm was applied to enhance native resolution (NR) images. NR and SR radiomics models were constructed, and the superior model was then integrated with clinical features to construct clinical-radiomics models. Diagnostic performance was evaluated by ROC analysis (AUC) and compared with radiologists’ assessments using the DeLong test.

**Results:**

A total of 486 male patients were enrolled for training (*n* = 338), domestic (*n* = 92), and international (*n* = 59) validation sets. The SR radiomics model achieved AUCs of 0.90, 0.82, and 0.91, respectively, in the training, domestic, and international validation sets, significantly surpassing the NR model (*p* < 0.001, *p* = 0.031, and *p* = 0.001, respectively). The clinical-radiomics model exhibited a significantly higher across both domestic and international validation sets compared to the SR radiomics model alone (0.95 vs 0.82, *p* = 0.004; 0.97 vs 0.91, *p* = 0.031). Moreover, the clinical-radiomics model surpassed the performance of experienced radiologists in both domestic (AUC, 0.95 vs 0.85, *p* = 0.012) and international (AUC, 0.97 vs 0.77, *p* < 0.001) validation cohorts.

**Conclusions:**

The SR-based clinical-radiomics model can effectively differentiate between SE and n-SE.

**Critical relevance statement:**

This international multicenter study demonstrated that a radiomics model of deep learning-based SR reconstructed US images enabled effective differentiation between SE and n-SE.

**Key Points:**

- Clinical parameters and radiologists’ assessments exhibit limited diagnostic accuracy for SE/n-SE differentiation in TGCT.
- Based on scrotal US images of TGCT, the SR radiomics models performed better than the NR radiomics models.
- The SR-based clinical-radiomics model outperforms both the radiomics model and radiologists’ assessment, enabling accurate, non-invasive preoperative differentiation between SE and n-SE.

**Graphical Abstract:**

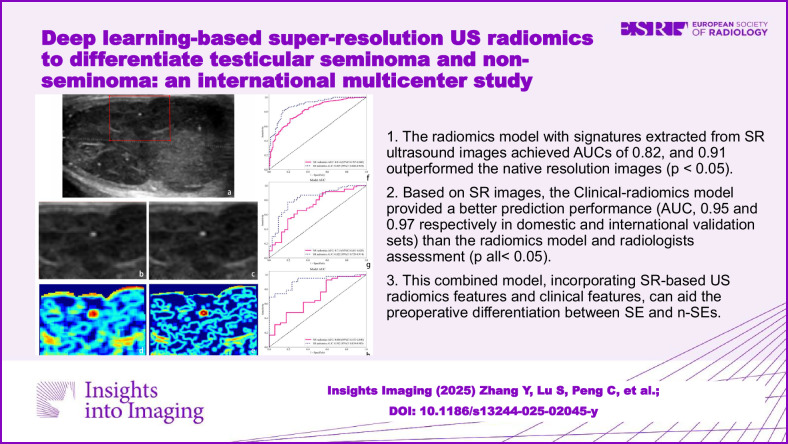

## Introduction

Testicular germ cell tumor (TGCT) comprise 95% [1] of testicular cancers and is morphologically categorized into seminoma (SE, 60%) and non-seminoma (n-SE, 40%) [2]. The progression rate of SE tends to be slower, with approximately 85% of SE initially diagnosed with clinical stage I disease as compared with 60% among n-SE [2]. Even within the same clinical stage, n-SE exhibit poorer prognosis than SE due to their distinct biological features [3]. Since n-SE has higher relapse rates [4] and susceptibility to retroperitoneal lymph node metastasis [5], prophylactic nerve-sparing retroperitoneal lymph node dissection (RPLND) is recommended even in clinical stage I, according to European Association of Urology [6] and European Society for Medical Oncology guidelines [7]. Given these differences in tumor biology, treatment, and prognosis, preoperative differentiation between SE and n-SE is imperative for clinical management decisions.

At present, the definitive diagnosis of TGCT depends upon pathology through inguinal orchidectomy [6] rather than scrotal biopsy because of the risk of tumor propagation [8]. The delay in obtaining a definitive pathological diagnosis in patients with n-SE necessitates a second-stage operation for RPLND, consequently prolonging their hospital stays and causing inconvenience. Hence, there is a strong need for noninvasive methods to distinguish TGCT subtypes pre-operatively, improving clinical management and patient compliance.

Tumor serum markers, including alpha-fetoprotein (AFP), beta-human chorionic gonadotropin (β-hCG), and lactate dehydrogenase (LDH), may hint at TGCT subtypes pre-operatively but have low sensitivity (SEN) [9]. Testicular ultrasound (US), employing high-frequency transducers (typically 7–15 MHz), provides superior spatial resolution for superficial testicular lesions compared to CT/MRI. This modality offers distinct advantages, including: (1) unparalleled soft-tissue contrast for testicular parenchyma, (2) procedural convenience with real-time imaging capability, and (3) complete absence of ionizing radiation exposure [10]. Therefore, testicular US is recommended as the first-line imaging modality for clinical diagnosis of TGCT. Some gray-scale US characteristics may indicate the subtype of TGCT, but are insufficient for reliable subtyping [11].

Radiomics offers a novel and effective method to probe image features invisible to human eyes. Recent studies have shown the potential of US radiomics in distinguishing malignancies in breast [12] and thyroid [13] tumors. Nevertheless, the diagnostic performance of native resolution (NR) US radiomics model could be compromised due to the low spatial resolution caused by the image reconstruction technique, improper depth settings, or focused error. Super-resolution (SR) reconstruction, based on mathematical or deep learning models, can augment spatial resolution from NR images [14]. Recently, deep learning-based SR techniques were successfully implemented in MRI and CT radiomics studies of breast, rectal, and lung cancers [15–17]. Similarly, SR has been used to enhance the resolution of 3D automated breast US, aiding in the differentiation of benign and malignant lesions [18]. However, the potential application and efficacy of SR in 2D ultrasound imaging remain unexplored.

This study aimed to explore the efficacy of a deep learning-based SR ultrasound radiomics model in differentiation between SE and n-SE.

## Methods

### Patients

This international multicenter retrospective study was approved by the institutional review boards at Sun Yat-sen University Cancer Center, and the requirement for informed consent was waived. This study was registered with the Chinese Clinical Trial Registry (ChiCTR2400092041). Patients who were diagnosed with definitive pathological TGCT subtypes and underwent testicular US examinations before surgery across nine Chinese hospitals and one Italian hospital from January 2015 to July 2023 were considered for inclusion. Exclusion criteria comprised: (1) patients under the age of 18; (2) previous treatment before US examinations; and (3) missing gray-scale US images or compromised image quality. The exclusion criteria resulted in 486 eligible patients with 489 lesions.

**Fig. 1:**
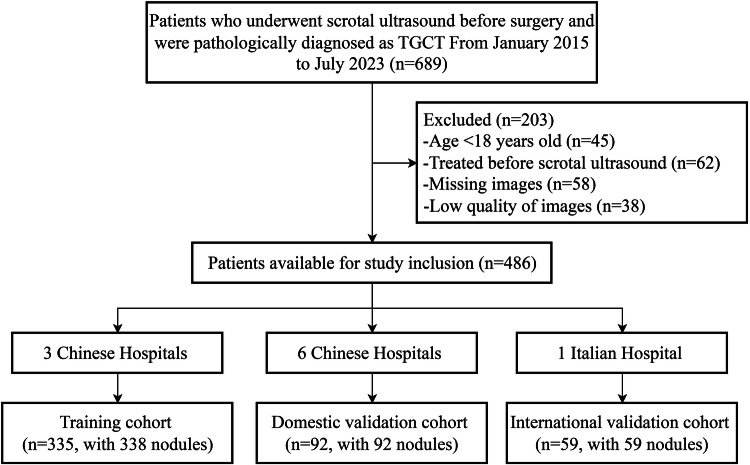
Patient recruitment flowchart. TGCT, testicular germ cell tumor; SE, seminoma; n-SE, non-seminoma

Patients from the three leading hospitals (Sun Yat-sen University Cancer Center, Fudan University Shanghai Cancer Center, and Henan Cancer Hospital) constituted our training cohort (*n* = 338), whilst participants from the six other Chinese hospitals (Zhejiang Cancer Hospital, Hunan Cancer Hospital, Yunnan Cancer Hospital, Sichuan Cancer Hospital, Linyi Cancer Hospital, and Zhuhai Chinese Medicine Hospital) served as the domestic validation group (*n* = 92). The international validation group enrolled patients from the University Hospital Trieste, Italy (*n* = 59). The patient recruitment flowchart is illustrated in Fig. 1.

### Data collection

The testicular US images were acquired from these 10 hospitals using multiple machines, including Siemens Acuson Sequoia 512 (Siemens Medical Solutions, Mountain View, CA), GE Logiq E9 (GE Healthcare), ARIETTA A60 (HITACHI), ESAOTE Mylab70 (ESAOTE), and so on. Scrotal US examinations were performed by trained radiologists (experience range: 5–15 years) using high-frequency linear transducers. Following complete bilateral evaluation, standardized images of detected testicular masses were obtained, including: the maximal tumor diameter plane, and orthogonal cross-sections. The plane with maximum diameter on the gray-scale US was retrieved.

Clinical data, including age at diagnosis, location, maximum tumor size, and tumor marker serology (AFP, LDH, and β-hCG), were collected from the electronic medical records of respective hospitals. The definitive TGCT subtype was determined by genitourinary pathologists through histopathological examination of surgical specimens.

### Deep learning based SR image reconstruction

**Fig. 2:**
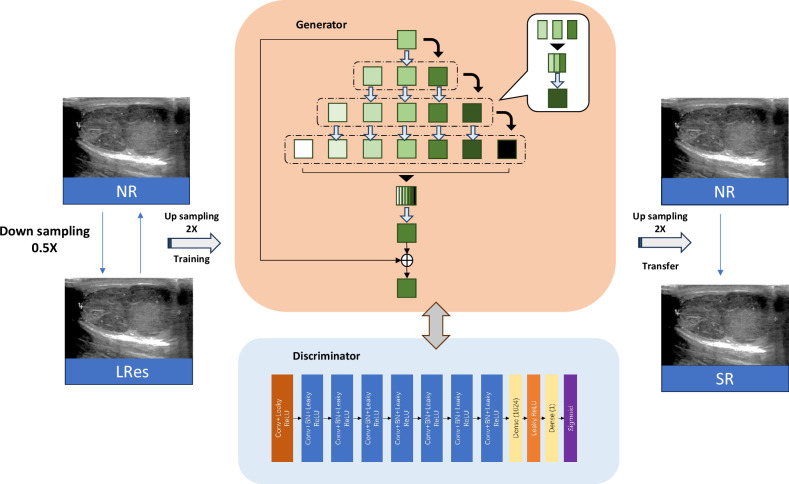
Deep learning-based super-resolution (SR) construction network. Firstly, Gaussian noise was introduced into native resolution (NR) images to generate novel low-resolution (LRes) images. Secondly, a generative adversarial network (GAN) model was then trained to learn the mapping between the paired LRes and NR images. Lastly, SR images were generated from NR images using the trained GAN model

The deep learning based SR image reconstruction model was provided by the OnekeyAI platform (https://github.com/OnekeyAI-Platform/onekey). This SR reconstruction technology has demonstrated commendable outcomes [19–21], resulting in a two-fold augmentation in the spatial resolution, while maintaining the original image size. Generative adversarial network (GAN) served as the basic architecture for this SR reconstruction technique. It involved two components- the generator and discriminator networks. The generator’s mission was to transform low-resolution images into high-resolution ones, while the discriminator’s task was to discern between genuine and artificial images. Both networks were trained in an adversarial manner, which helped the generator network hone its capacity to transpose low to high-resolution images. The SR reconstruction process was illustrated in Fig. 2, with comprehensive elucidations offered in the Supplementary Material.

Peak signal-to-noise ratio (PSNR) and structure similarity index metric (SSIM) were calculated for the evaluation of SR performance in this study.

### Segmentation, feature extraction, and feature selection

The regions of interest (ROIs) for tumor segmentation were manually delineated on both NR and SR ultrasound images by a radiologist with 5 years of experience (Y.Z.), blinded to the clinical information, using 3D Slicer version 5.6.1 (https://www.slicer.org) and subsequently reviewed by another radiologist with 15 years of experience (J.Z.) to ensure accuracy. The ROI identified will be stored in NIfTI (NII) format. Subsequently, the Pyradiomics software package (version 3.1.0) operating on Python (version 3.7) was utilized for feature extraction. The radiomics features, including three types: geometry, intensity, and texture, were extracted from the NR and SR images. Geometry describes the shape features of the tumor, while intensity considers the first-order statistical distribution of the pixels within the tumor. Texture features represent the patterns or secondary/ higher order spatial distributions of the intensities, and they were typically extracted via different methods, such as gray-level co-occurrence matrix (GLCM), gray-level run length matrix (GLRLM), gray-level size zone matrix (GLSZM), and neighborhood gray-tone difference matrix (NGTDM).

For reproducibility assessment, 80 randomly-selected cases were independently segmented by two radiologists (Y.Z. and S.L., with 5 years and 2 years of scrotal US experience, respectively). Radiomics features with intra-class correlation coefficients (ICC) ≤ 0.90 across readers were excluded to ensure analysis robustness.

To mitigate the redundant features, the subsequent step was feature selection. First, a univariate Student’s *t*-test was conducted to screen for the extracted features, and those with a *p* value < 0.05 were kept. Then, the Pearson correlation coefficient was calculated between features. And one of the features with a correlation coefficient greater than 0.9 between any two features is retained to reduce redundancy. Finally, the least absolute shrinkage and selection operator (LASSO) regression algorithm with 5-fold cross-validation was employed to identify the most relevant features. The retained features were then used to construct a radiomics model. The complete flow of feature selection can be seen in Fig. S1a.

### NR and SR radiomics model establishment and comparison

Seven unique machine learning methodologies, including logistic regression (LR), support vector machine (SVM), K-nearest neighbors (KNN), RandomForest, extremely randomized trees (ExtraTrees), extreme gradient boosting (XGBoost), and light gradient boosting machine (LightGBM), were utilized for the construction of both NR and SR radiomic models. The 5-fold cross-validation methodology was implemented to determine the optimal machine learning technique. Then, the effectiveness of NR and SR radiomics models was compared to determine the final radiomics model.

### Comparison of diagnostic performance between radiomics, clinical, and clinical-radiomics models

Univariate and multivariate LR analysis was executed on the clinical data, discerning those exhibiting a *p* value of less than 0.05 for selection into the clinical model. Post this, the odds ratio (OR) with a 95% confidence interval (CI) was calculated. Subsequently, a hybrid clinical-radiomics model was proposed to incorporate statistically pivotal clinical indicators along with the selected radiomics model. The performances of radiomics, clinical, and clinical-radiomics models were assessed in training, domestic, and international validation sets to ascertain the optimal model.

### Image analysis of radiologists

**Fig. 3:**
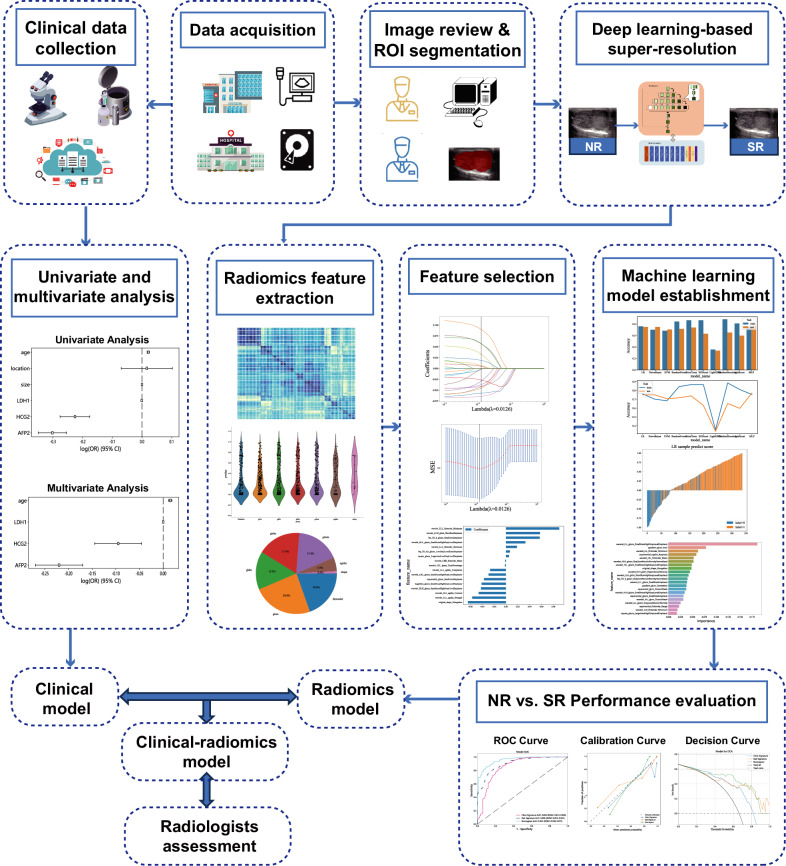
Workflow for construction of clinical model, deep-learning based SR reconstruction, feature extraction and selection, construction of radiomics model, and clinical-radiomics model. ROI, region of interest; NR, native resolution; SR, super-resolution

According to a world federation for ultrasound in medicine and biology position paper about testis [22], US images of SE were more commonly homogenous, hypoechoic, and lobulated, while images of n-SE were more often heterogenous with cystic or calcified components. Testicular US images and laboratory data in the domestic and validation datasets were reviewed by two experienced radiologists (P.C. and Z.W., with over 10 years of experience in testicular US) who were blinded to the pathological data. SE, n-SE, or uncertainty was assigned for each patient. Disagreement between the two readers was resolved by joint discussion. Figure 3 demonstrates the schematic of the whole study.

### Statistical analysis

Categorical variables were presented as counts with percentages and compared using the Chi-square test or the Kruskal–Wallis *H*-test. Continuous variables were presented as medians with interquartile ranges and analyzed using the Kruskal–Wallis *H*-test. Diagnostic performances were evaluated by the area under the receiver operating characteristic curve (AUC), accuracy, SEN, specificity (SPE), positive predictive value (PPV), and negative predictive value (NPV). The Delong method was adapted to compare the AUC values. A *p* value < 0.05 was deemed statistically significant. Decision curve analysis (DCA) was then conducted for the evaluation of the clinical utility of different diagnostic models. The calibration curves were used to evaluate the consistency between the actual and predicted TGCT subtypes. All statistical analyses were conducted using Python (version 3.7) and SPSS (version 26.0).

## Results

### Clinical characteristics

A total of 486 patients with 489 testicular lesions were enrolled in this study, comprising 321 SEs (65.6%) and 168 n-SEs (34.4%). The lesions were then split for model development as follows: a training cohort (*n* = 338, 64.5% as SE), a domestic validation cohort (*n* = 92, 66.3% as SE), and an international validation cohort (*n* = 59, 71.2% as SE). The clinical baseline characteristics of three datasets are shown in Table 1.

**Table 1:** Clinical and pathological characteristics of patients in the training, domestic, and international validation cohorts

Characteristic	Training cohort (*n* = 338)	Domestic validation cohort (*n* = 92)	International validation cohort (*n* = 59)	*p* value
Age (years)*	33 (27–38)	35 (29–43)	34 (28–42)	0.012^†^
Maximum diameter (mm)				0.032^†^
≤ 25	67 (19.8)	16 (17.4)	20 (33.9)	
> 25	271 (80.2)	76 (82.6)	39 (66.1)	
Location				0.852
Right	168 (49.7)	44 (47.8)	31 (52.5)	
Left	170 (50.3)	48 (52.2)	28 (47.5)	
Histological subtypes				0.601
SE	218 (64.5)	61 (66.3)	42 (71.2)	
n-SE	120 (35.5)	31 (33.7)	17 (28.8)	
LDH (ng/mL)*	207.0 (167.0–305.8)	266.7 (182.7–305.8)	205.0 (176.0–246.0)	0.044^†^
AFP (ng/mL)				0.233^†^
≤ 8	248 (73.4)	64 (69.6)	48 (81.4)	
8–40	19 (5.6)	12 (13.0)	6 (10.2)	
> 40	71 (21.0)	16 (17.4)	5 (8.5)	
HCG (ng/mL)				0.014^†^
≤ 5	205 (60.7)	58 (63.0)	47 (79.7)	
5–200	78 (23.1)	22 (23.9)	9 (15.3)	
> 200	55 (16.3)	12 (13.0)	3 (5.1)	

Unless otherwise specified, data are numbers of patients, with percentages in parentheses

*SE* seminoma, *n-SE* non-seminoma, *LDH* lactate dehydrogenase, *AFP* alpha-fetoprotein, *HCG* human chorionic gonadotropin

* Data are medians; data in parentheses are the interquartile range

^†^ Kruskal–Wallis *H*-test was performed

Multivariable LR analysis showed that individuals with SE had older age and a lower level of serum AFP, LDH, and HCG (*p* < 0.05) (Table 2). These four clinically significant variables were subsequently chosen for the construction of the clinical model.

**Table 2:** Uni- and multivariable LR analysis of clinical variables

Clinical variables	Univariable analysis			Multivariable analysis		
	OR	95% CI	*p* value	OR	95% CI	*p* value
Age	1.13	1.097–1.164	< 0.001	1.106	1.064–1.149	< 0.001
Location						
Right	Ref.					
Left	1.057	0.727–1.535	0.773	…	…	…
Size (mm)						
≤ 25	Ref.					
> 25	0.979	0.619–1.549	0.928	…	…	…
LDH (ng/mL)	0.999	0.999–1.000	0.016	1.012	1.001–1.003	0.012
AFP (ng/mL)						
≤ 8	Ref.			Ref.		
8–40	0.149	0.074–0.303	< 0.001	0.125	0.053–0.292	< 0.001
> 40	0.009	0.003-0.025	< 0.001	0.012	0.004–0.038	< 0.001
HCG (ng/mL)						
≤ 5	Ref.			Ref.		
5–200	0.296	0.186–0.472	< 0.001	0.591	0.311–1.121	0.107
> 200	0.083	0.045–0.154	< 0.001	0.18	0.076–0.428	<0.001

*OR* odds ratio, *CI* confidence interval, *LDH* lactate dehydrogenase, *AFP* alpha-fetoprotein, *HCG* human chorionic gonadotropin

### Performance of SR vs NR radiomics models

**Fig. 4:**
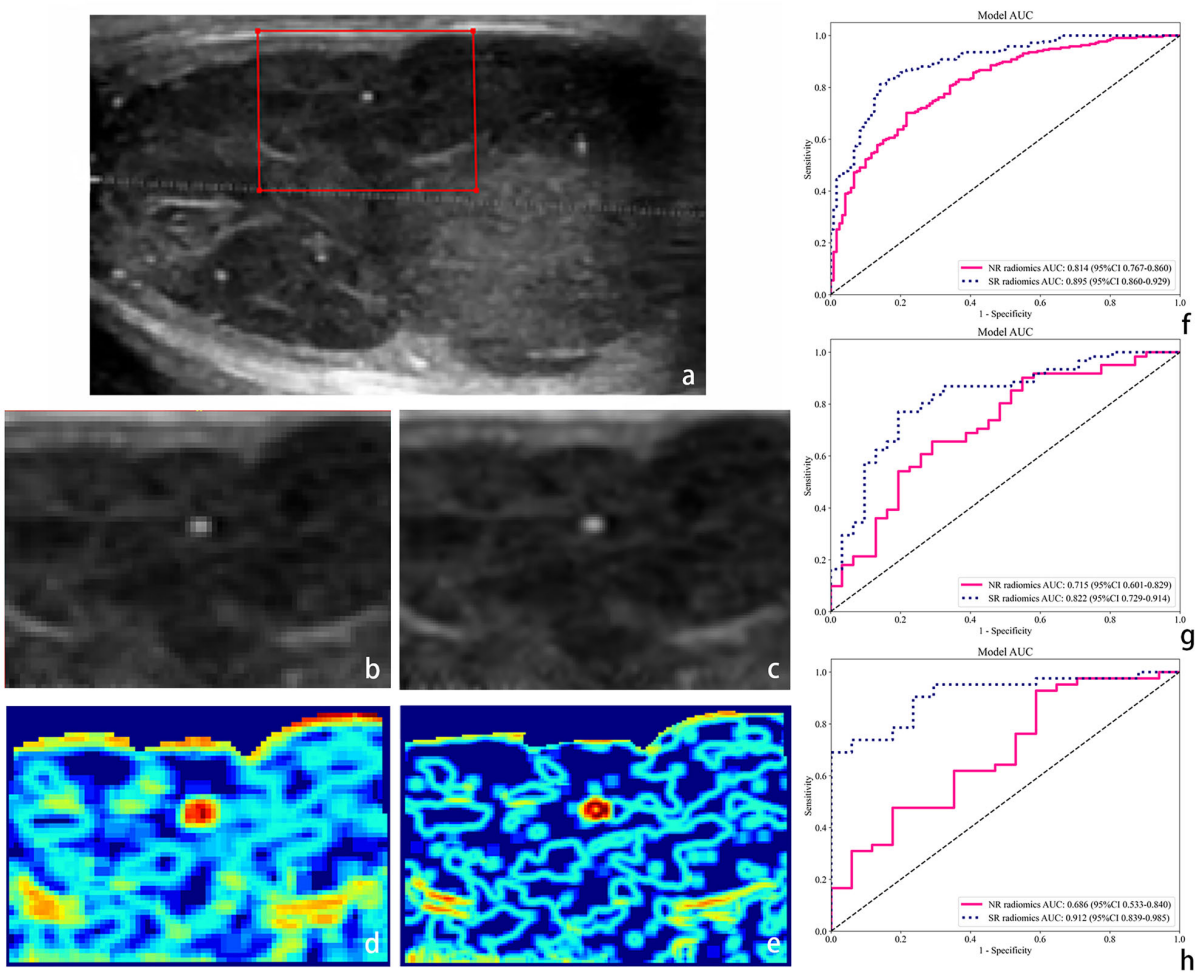
Influence of SR reconstruction of US images on the performance of radiomics models. **a** US image at NR; **b**, **c** the improvements in image quality achieved by SR reconstruction. **d**, **e** First-order entropy evaluation of images with different resolutions. **f**–**h** ROC of radiomics models based on different spatial resolutions of the training, domestic, and international validation cohorts, respectively. SR radiomics model had significantly higher AUCs than those in the NR model (*p* < 0.001, *p* = 0.031, *p* = 0.001, respectively)

After SR reconstruction, the US image quality was significantly improved (Fig. 4a–e), and the PSNR was 44.06 ± 4.11 dB and the SSIM was 0.9967 ± 0.0065.

Figure S1b, c display the extracted radiomic features for NR and SR images. Although there was partial overlap in the selected features, not all were common to both. The LR machine learning algorithm, which exhibited superior performance in the 5-fold cross-validation, was chosen for the construction of NR and SR radiomics models (Fig. S2). In comparison to the NR radiomics model, the SR radiomics model showed significantly superior AUC in the training (0.90 vs 0.81, *p* < 0.001), domestic validation (0.82 vs 0.72, *p* = 0.031) and international validation (0.91 vs 0.69, *p* = 0.001) cohorts (Fig. 4f–h), and was selected as the final radiomics model.

### Performance of the clinical-radiomics model vs the clinical model, the radiomics model, and the radiologists’ assessment

**Fig. 5:**
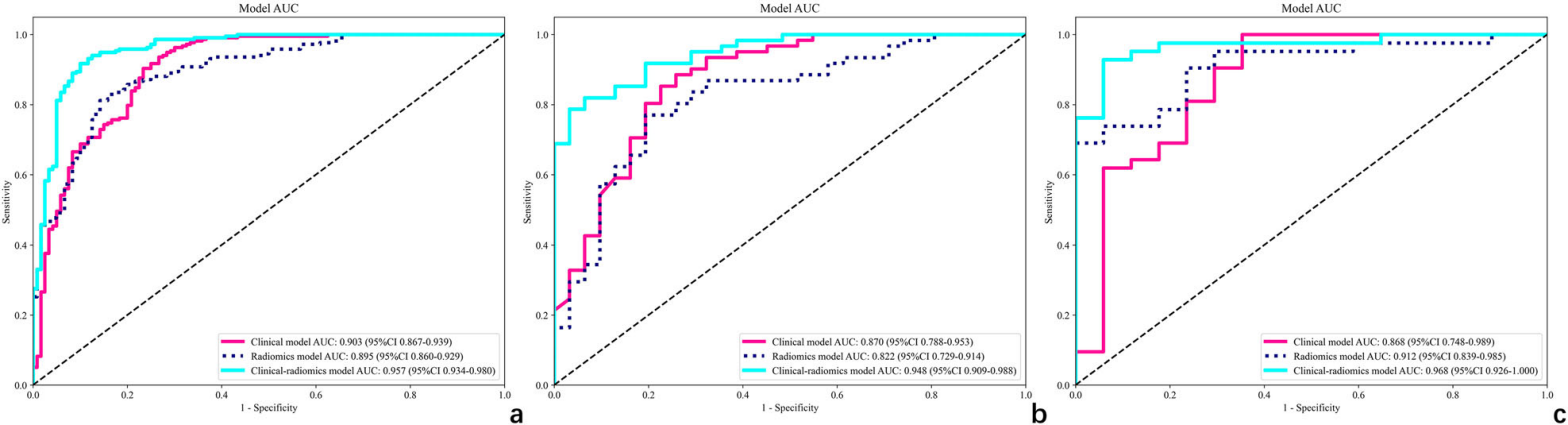
ROC for clinical, radiomics, and clinical-radiomics combined models in the training (**a**), domestic (**b**), and international (**c**) validation cohorts

As illustrated in Fig. 5, the clinical-radiomics model demonstrated more superior discriminatory capability for differentiating TGCT subtypes than clinical model in the domestic validation cohort (AUC: 0.95 vs 0.87, *p* = 0.038), and radiomics models in both the domestic (AUC: 0.82, *p* = 0.004) and international validation cohort (AUC: 0.97 vs 0.91, *p* = 0.031). The AUC of the clinical-radiomics model was higher than that of the clinical model in the international validation cohort (0.97 vs 0.87), although the difference was not significant (*p* = 0.099). Calibration curves showed good calibration of the projected endpoints and actual values. The clinical-radiomics model achieved the largest benefit compared to the other two models when the threshold probability ranges from 0.43 to 0.96 in the domestic validation cohort and 0.45 to 0.84 in the international validation cohort (Fig. S3).

For the comparison with experienced radiologists, the AUC of the radiomics model did not significantly differ from that of radiologists’ assessment (0.82 vs 0.85, *p* = 0.670) in the domestic validation cohort, while in the international validation cohort, the AUC of the radiomics model surpassed that of radiologists’ assessment (0.91 vs 0.77, *p* = 0.036). After incorporation of clinical variables, the clinical-radiomics model showed significantly higher AUCs than the radiologists’ assessments in both domestic and international validation cohorts (*p* = 0.012 and *p* < 0.001, respectively) (Table 3).

**Table 3:** Performance of radiomics, clinical, and clinical-radiomics models, as well as radiologists’ assessment

Dataset	AUC	SEN (%)	SPE (%)	PPV (%)	NPV (%)	p^*^ value	*p*^†^ value
Domestic validation cohort							
Clinical model	0.87 [0.79, 0.95]	86.9 (53/61) [80.0, 93.8]	74.2 (23/31) [65.3, 83.1]	86.9 (53/61) [80.0, 93.8]	74.2 (23/31) [65.3, 83.1]	0.038	0.679
Radiomics model	0.82 [0.73, 0.91]	75.4 (46/61) [66.6, 84.2]	80.6 (25/31) [72.6, 88.7]	88.5 (46/52) [81.9, 95.0]	62.5 (25/40) [52.6, 72.4]	0.004	0.670
Clinical-radiomics model	0.95 [0.91, 0.99]	80.3 (49/61) [72.2, 88.5]	93.5 (29/31) [88.5, 98.6]	96.1 (49/51) [92.1, 100.0]	70.7 (29/41) [61.4, 80.0]	…	0.012
Radiologists’ assessment	0.85 [0.75, 0.94]	80.3 (49/61) [72.2, 88.5]	83.9 (26/31) [76.4, 91.4]	90.7 (49/54) [84.8, 96.7]	68.4 (26/38) [58.9, 77.9]	0.012	…
International validation cohort							
Clinical model	0.87 [0.75, 0.99]	97.6 (41/42) [93.7, 101.5]	64.7 (11/17) [52.5, 76.9]	87.2 (41/47) [78.7, 95.7]	91.7 (11/12) [84.6, 98.7]	0.099	0.200
Radiomics model	0.91 [0.84, 0.98]	66.7 (28/42) [54.6, 78.7]	100.0 (17/17) [100.0,100.0]	100.0 (28/28) [100.0, 100.0]	54.8 (17/31) [42.1, 67.5]	0.031	0.036
Clinical-radiomics model	0.97 [0.93, 1.00]	90.5 (38/42) [83.0, 98.0]	94.1 (16/17) [88.1, 100.1]	97.4 (38/39) [93.4,101.5]	80.0 (16/20) [69.8, 90.2]	…	< 0.001
Radiologists’ assessment	0.77 [0.63, 0.90]	66.7 (28/42) [54.6, 78.7]	82.4 (14/17) [72.6, 92.1]	90.3 (28/31) [82.8, 97.9]	50.0 (14/28) [37.2, 62.8]	< 0.001	…

Data in parentheses are the number of patients, and data in brackets are 95% CIs

*AUC* area under the curve, *NPV* negative predictive value, *PPV* positive predictive value

* *p* values indicate the significance level of the comparison of AUCs with the clinical-radiomics combined model in the corresponding cohorts by using the DeLong test

^†^ *p* values indicate the significance level of the comparison of AUCs with the radiologist assessment in the corresponding cohorts by using the DeLong test

## Discussion

Accurate preoperative differentiation between SE and n-SE remains clinically challenging in TGCT, as conventional diagnostic approaches—including clinical parameters and radiologic assessment—demonstrate limited discriminatory capability. Testicular US, remains the frontline imaging for testicular mass evaluation; however, its diagnostic resolution could suffer from image reconstruction techniques and operator-dependent variability. In this study, based on testicular US images, a pre-trained deep-learning-based SR radiomics model was applied to enhance the NR images. The SR radiomics models performed better than the NR radiomics models. The SR-based clinical-radiomics model (0.95 and 0.97, respectively) outperforms both the radiomics model and the radiologists’ assessment in both domestic and international validation cohorts (*p* < 0.05 for all), enabling accurate, non-invasive preoperative differentiation between SE and n-SE.

When trained on clinical parameters only, the clinical model achieved an AUC of 0.87 in both domestic and international validation cohorts. This corresponds to the different expression of tumor serum markers, including LDH, β-hCG, and AFP, in SE and n-SE. AFP does not occur in pure SE by definition [23] while often elevated in 90% of n-SE patients [24]. Approximately 15% of SE [25] and 90% of n-SE [24] patients will have elevated β-hCG levels. LDH serves as a tumor mass marker and an adverse prognostic factor, but is not a specific marker in differentiating TGCT subtypes [26]. The measurement of serum markers is crucial for the initial evaluation of patients with testicular tumors, but elevated serum markers alone do not provide a definitive diagnosis. The application of tumor markers may be constrained by the poor SPE (74.2% and 64.7%, respectively, in domestic and international validation cohorts), thereby underscoring the need for more robust and accurate diagnostic tools.

High-frequency US is recommended as the first-line imaging modality by clinical guidelines to preoperatively characterize testicular tumors [6, 7]. Previous studies showed that solid components were associated with SE while cystic changes were related to n-SE, however, the SEN was only 76.8% and 57.7%, respectively [27]. Our study corroborates these findings in radiologists’ assessments, demonstrating similar performance limitations. However, diagnostic accuracy based solely on subjective imaging interpretation may not fully meet clinical requirements for optimal patient management.

To address these limitations, we developed a novel US radiomics model. It is important to note that radiomics model performance is highly dependent on image quality, as low spatial resolution may compromise feature extraction and analytical accuracy. Therefore, we implemented an established SR technique, which is based on GAN technology, to enhance the resolution of testicular US images. GANs can generate highly realistic, detailed, high-resolution images by effectively restoring high-frequency detail information [28]. Compared to conventional interpolation and reconstruction methods, GAN-generated images exhibit substantially reduced distortion and blurring during magnification, leading to marked improvements in image quality [29, 30]. In our study, both qualitative visual assessment and quantitative PSNR measurements demonstrated significant image quality improvement following SR processing. Consistent with previous SR studies [31], the feature selection process yielded different discriminative features after SR reconstruction, ultimately resulting in superior performance of the SR model compared to the NR model, which indicates the need for improvement before clinical implementation.

The superior diagnostic performance of the SR-based clinical-radiomics model derives from two key factors: (1) enhanced resolution from SR that improves feature extraction, and (2) strong correlations between radiomic features and underlying histopathology. Histologically, SE consists of relatively homogeneous primordial germ cells or gonocytes, while n-SE demonstrates diverse differentiation patterns from embryonic and extra-embryonic tissues [7, 32]. Consequently, radiomic features can effectively quantify texture information to characterize the intratumoral heterogeneity of tissue components in TGCT, thereby enabling reliable differentiation between SE and n-SE subtypes.

Neither CT nor MRI demonstrates superior diagnostic performance for evaluating superficial testicular masses. These modalities primarily serve complementary roles in clinical staging, particularly for assessing retroperitoneal lymph node metastasis and distant organ involvement. Consequently, most patients with early-stage testicular tumors do not routinely undergo CT or MRI examinations. Although several preliminary studies have reported the potential of CT- and MRI-based radiomics for distinguishing SE from non-SE, the clinical validity of these findings remains questionable due to limited sample sizes [32–35]. These methodological limitation underscores the comparative strength of our study.

The present study has certain limitations. Firstly, as a retrospective multicenter study, the US images were acquired using different devices across institutions. The consistency of radiomic features across different devices was not feasible in this study due to its retrospective design. Therefore, future prospective studies are necessary for broader clinical application. Secondly, while our sample size represents the largest multicenter cohort in published testicular ultrasound radiomics studies to date, the absolute number remains modest due to the inherent rarity of testicular tumors.

The deep learning-based SR reconstruction was an effective method to enhance testis US quality, as well as the radiomics feature capability in discerning between TGCT subtypes. The SR clinical-radiomics model demonstrated excellent performance and the potential to be implemented in a clinical setting as a preoperative diagnostic tool for patients with TGCT.

## Supplementary information

ELECTRONIC SUPPLEMENTARY MATERIAL

## Data Availability

The data analyzed in this research can be obtained from the corresponding author on reasonable request approved by the institutional review board of Sun Yat-sen University Cancer Center.

## Abbreviations

AFP: Alpha-fetoprotein
AUC: Area under the curve
β-hCG: Beta-human chorionic gonadotropin
CI: Confidence interval
GAN: Generative adversarial network
LDH: Lactate dehydrogenase
LR: Logistic regression
NR: Native resolution
NPV: Negative predictive value
n-SE: Non-seminoma
OR: Odds ratio
PSNR: Peak signal-to-noise ratio
PPV: Positive predictive value
RPLND: Prophylactic nerve-sparing retroperitoneal lymph node dissection
ROC: Receiver operating characteristic curves
ROIs: Regions of interest
SE: Seminoma
SEN: Sensitivity
SPE: Specificity
SSIM: Structure similarity index metric
SR: Super-resolution
TGCT: Testicular germ cell tumor
