# Supplementary material for: Deep learning-based super-resolution US radiomics to differentiate testicular seminoma and non-seminoma: an international multicenter study
Source: Insights Imaging. 2025 Aug 1;16:165. doi: 10.1186/s13244-025-02045-y (PMC12316629; doi:10.1186/s13244-025-02045-y)
Supplement: Supplementary file 1 — ELECTRONIC SUPPLEMENTARY MATERIAL [file 13244_2025_2045_MOESM1_ESM.pdf]

Deep learning-based super-resolution US radiomics to differentiate testicular seminoma and non-seminoma: an international multicenter study

ELECTRONIC SUPPLEMENTARY MATERIAL

The datasets utilized for training the SR reconstruction technique comprised an extensive of images. These images were separated into low- and high-resolution image sets, with low-resolution images created via downsampling from their original high- resolution counterparts.The loss function used in the GAN model encompassed three aspects: gradient loss, L1 loss(21), and perceptual loss(22). Gradient loss encouraged the generated images to have similar gradient values to the high-resolution images. L1 loss measured the pixel-by-pixel discrepancy between the high-resolution and generated images while the perceptual loss evaluated the dissimilarity in the feature representations between them. The combination of these loss functions helped to ensure that the generated images were visually similar to the high-resolution images. The matched pair datasets were used to train the GAN model, which was conducted on the Onekey AI platform.

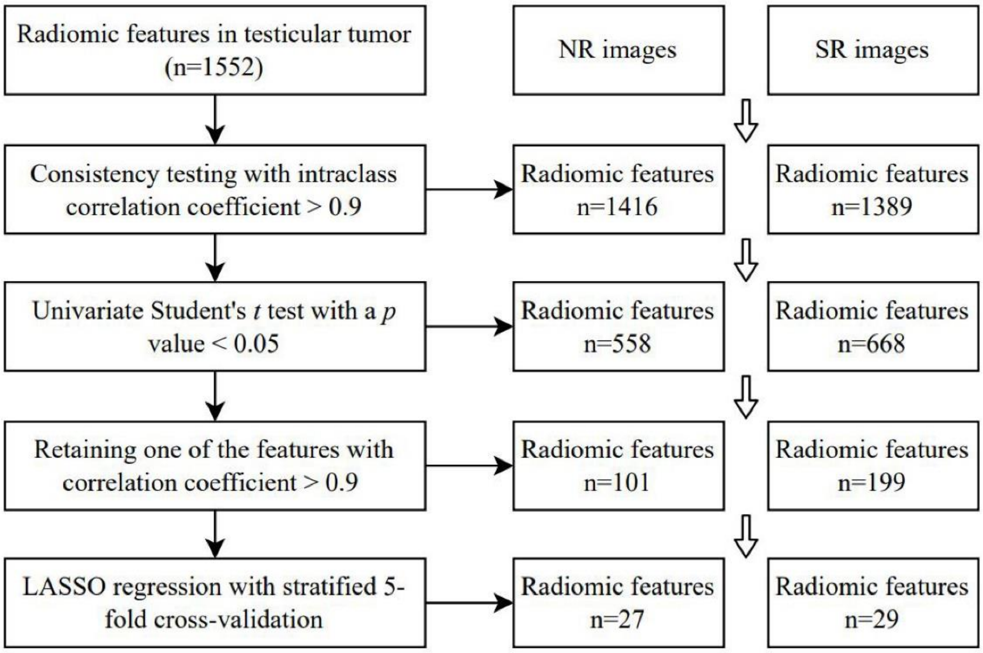
Radiomic features in testicular tumor (n=1552)
NR images
SR images
Consistency testing with intraclass correlation coefficient > 0.9
Radiomic features n=1416
Radiomic features n=1389
Univariate Student's t test with a p value < 0.05
Radiomic features n=558
Radiomic features n=668
Retaining one of the features with correlation coefficient > 0.9
Radiomic features n=101
Radiomic features n=199
LASSO regression with stratified 5-fold cross-validation
Radiomic features n=27
Radiomic features n=29

a

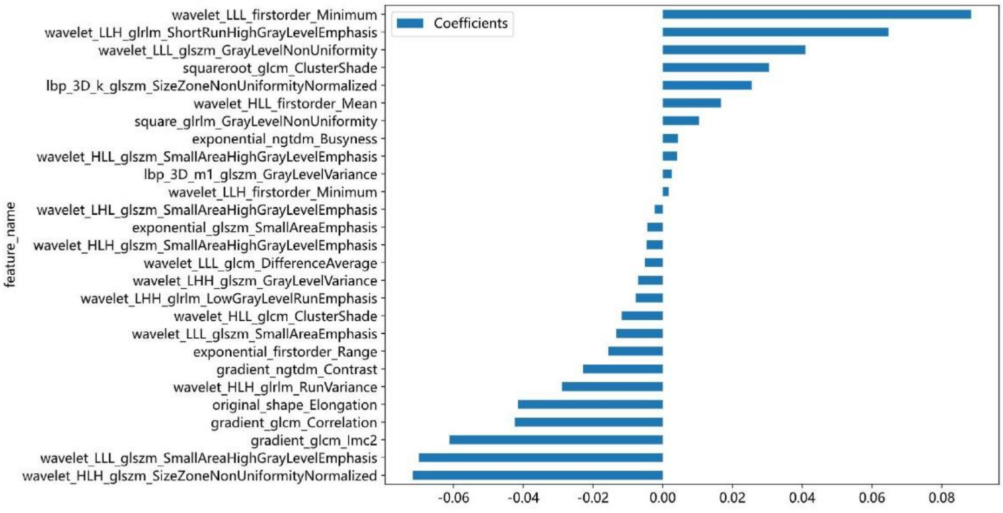
Coefficients
feature_name
wavelet_LLL_firstorder_Minimum
wavelet_LLH_glrlm_ShortRunHighGrayLevelEmphasis
wavelet_LLL_glszm_GrayLevelNonUniformity
squareroot_glcm_ClusterShade
lbp_3D_k_glszm_SizeZoneNonUniformityNormalized
wavelet_HLL_firstorder_Mean
square_glrlm_GrayLevelNonUniformity
exponential_ngtdm_Busyness
wavelet_HLL_glszm_SmallAreaHighGrayLevelEmphasis
lbp_3D_m1_glszm_GrayLevelVariance
wavelet_LLH_firstorder_Minimum
wavelet_LHL_glszm_SmallAreaHighGrayLevelEmphasis
exponential_glszm_SmallAreaEmphasis
wavelet_HLH_glszm_SmallAreaHighGrayLevelEmphasis
wavelet_LLL_glcm_DifferenceAverage
wavelet_LHH_glszm_GrayLevelVariance
wavelet_LHH_glrlm_LowGrayLevelRunEmphasis
wavelet_HLL_glcm_ClusterShade
wavelet_LLL_glszm_SmallAreaEmphasis
exponential_firstorder_Range
gradient_ngtdm_Contrast
wavelet_HLH_glrlm_RunVariance
original_shape_Elongation
gradient_glcm_Correlation
gradient_glcm_Imc2
wavelet_LLL_glszm_SmallAreaHighGrayLevelEmphasis
wavelet_HLH_glszm_SizeZoneNonUniformityNormalized
-0.06
-0.04
-0.02
0.00
0.02
0.04
0.06
0.08

b

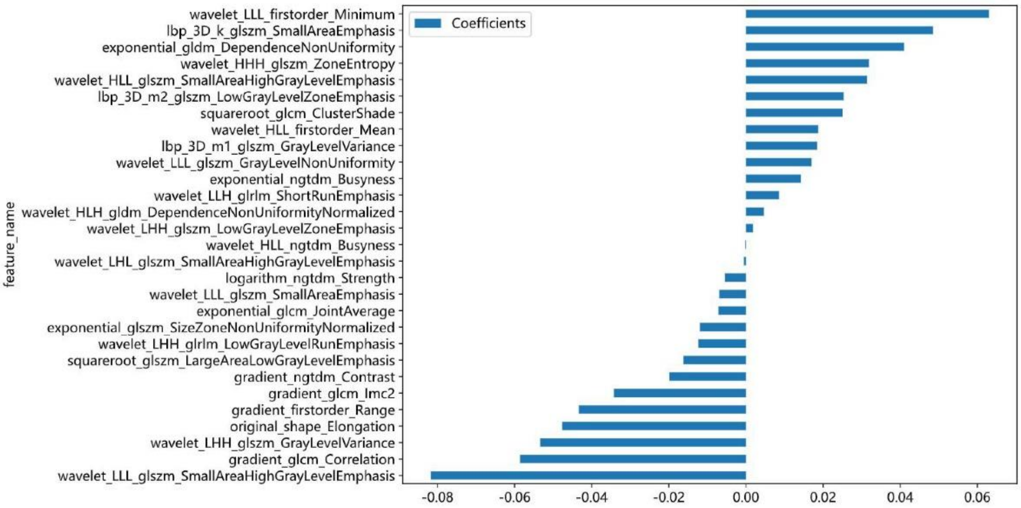
Coefficients
feature_name
wavelet_LLL_firstorder_Minimum
lbp_3D_k_glszm_SmallAreaEmphasis
exponential_gldm_DependenceNonUniformity
wavelet_HHH_glszm_ZoneEntropy
wavelet_HLL_glszm_SmallAreaHighGrayLevelEmphasis
lbp_3D_m2_glszm_LowGrayLevelZoneEmphasis
squareroot_glcm_ClusterShade
wavelet_HLL_firstorder_Mean
lbp_3D_m1_glszm_GrayLevelVariance
wavelet_LLL_glszm_GrayLevelNonUniformity
exponential_ngtdm_Busyness
wavelet_LLH_glrlm_ShortRunEmphasis
wavelet_HLH_gldm_DependenceNonUniformityNormalized
wavelet_LHH_glszm_LowGrayLevelZoneEmphasis
wavelet_HLL_ngtdm_Busyness
wavelet_LHL_glszm_SmallAreaHighGrayLevelEmphasis
logarithm_ngtdm_Strength
wavelet_LLL_glszm_SmallAreaEmphasis
exponential_glcm_JointAverage
exponential_glszm_SizeZoneNonUniformityNormalized
wavelet_LHH_glrlm_LowGrayLevelRunEmphasis
squareroot_glszm_LargeAreaLowGrayLevelEmphasis
gradient_ngtdm_Contrast
gradient_glcm_Imc2
gradient_firstorder_Range
original_shape_Elongation
wavelet_LHH_glszm_GrayLevelVariance
gradient_glcm_Correlation
wavelet_LLL_glszm_SmallAreaHighGrayLevelEmphasis
-0.08
-0.06
-0.04
-0.02
0.00
0.02
0.04
0.06

c

Figure S1. Complete workflow (a) and results (b, c) of feature selection. Feature weight distribution plot for NR (b) and SR (c) images presents the extracted features with corresponding coefficient values.

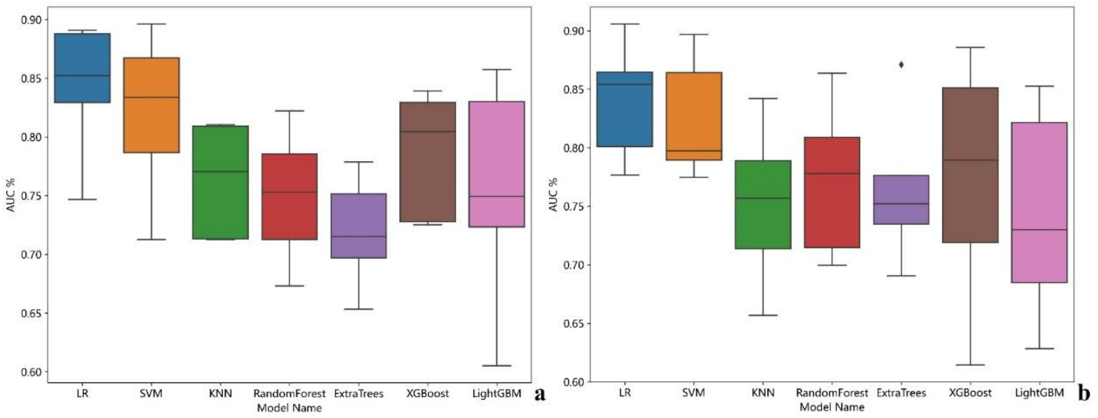

Figure S2. Comparison of area under the receiver operating characteristic curves across different machine learning algorithms-based models and super-resolution levels including native-resolution (a) and super-resolution (b).

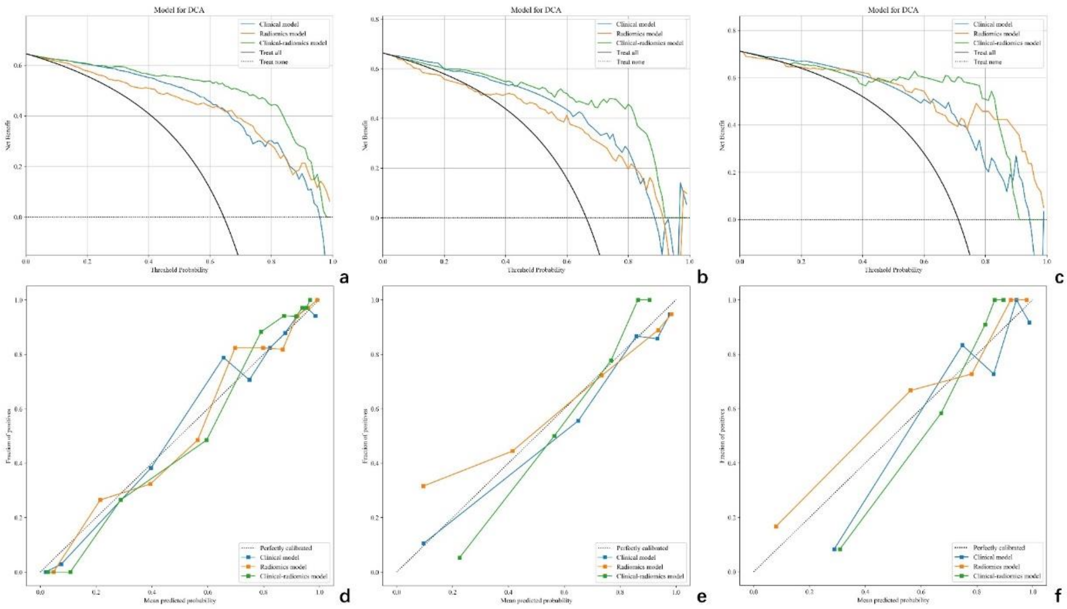

Figure S3. Decision curve analysis in the training (a), domestic (b), and international (c) validation cohort. Calibration curves of the training (d), domestic (e) and international (f) validation cohort, respectively.
